# Improved perioperative narcotic usage patterns in patients undergoing robotic-assisted compared to manual total hip arthroplasty

**DOI:** 10.1186/s42836-023-00211-5

**Published:** 2023-11-04

**Authors:** Graham B. J. Buchan, Zachary Bernhard, Christian J. Hecht, Graeme A. Davis, Trevor Pickering, Atul F. Kamath

**Affiliations:** 1 Cleveland Clinic Orthopaedic and Rheumatologic Institute, 9500 Euclid Avenue, Cleveland, OH 44195 USA; 2 Pinehaven Orthopaedic and Arthroplasty Institute, Krugersdorp, Johannesburg, 1739 South Africa; 3 Life Wilgeheuwel Hospital, Roodepoort, Johannesburg, 1724 South Africa; 4 Mississippi Sports Medicine and Orthopaedic Center, Jackson, MS 39202 USA

**Keywords:** Robotic-assisted total hip arthroplasty, Narcotics, Opioids, Outcomes

## Abstract

**Background:**

Robot-assisted total hip arthroplasty (RA-THA) improves accuracy in achieving the planned acetabular cup positioning compared to conventional manual THA (mTHA), but optimal dosage for peri-RA-THA and mTHA pain relief remains unclear. This study aimed to compare pain control with opioids between patients undergoing direct anterior approach THA with the use of a novel, fluoroscopic-assisted RA-THA system compared to opioid consumption associated with fluoroscopic-assisted, manual technique.

**Methods:**

Retrospective cohort analysis was performed on a consecutive series of patients who received mTHA and fluoroscopy-based RA-THA. The average amount of postoperative narcotics in morphine milligram equivalents (MME) given to each cohort was compared, including during the in-hospital and post-discharge periods. Analyses were performed on the overall cohort, as well as stratified by opioid-naïve and opioid-tolerant patients.

**Results:**

The RA-THA cohort had significantly lower total postoperative narcotic use compared to the mTHA cohort (103.7 vs. 127.8 MME; *P* < 0.05). This difference was similarly seen amongst opioid-tolerant patients (123.6 vs. 181.3 MME; *P* < 0.05). The RA-THA cohort had lower total in-hospital narcotics use compared to the mTHA cohort (42.3 vs. 66.4 MME; *P* < 0.05), consistent across opioid-naïve and opioid-tolerant patients. No differences were seen in post-discharge opioid use between groups.

**Conclusions:**

Fluoroscopy-based RA-THA is associated with lower postoperative opioid use, including during the immediate perioperative period, when compared to manual techniques. This may have importance in rapid recovery protocols and mitigating episode burden of care.

## Background

One of the largest challenges facing contemporary arthroplasty surgeons is balancing the need for improved perioperative pain management while practicing good opioid stewardship [1]. While effective pain control in the post-arthroplasty patient is an important measure of improvement and has been associated with increased postoperative mobility and recovery [2–4], joint arthroplasty, including total hip arthroplasty (THA), has been associated with relatively high postoperative opioid prescriptions given deep dissection, joint arthrotomy, and bone cuts [5].

Postoperative pain control can be improved by using new surgical techniques, multimodal pain approaches, new prescribing methods, and the integration of robot-assisted technologies [1, 6–9]. However, despite these advances, opioids remain a mainstay in postoperative pain control [1, 10, 11]. The use of robotic arthroplasty systems has been shown to improve postoperative pain and reduce narcotic burden compared to manual techniques [12–15]. While the use of robotic-assisted technologies for THA has expanded rapidly given the promise of increased precision of acetabular cup placement [16], a paucity exists in the literature comparing opioid prescribing and consumption patterns surrounding robotic-assisted THA (RA-THA).

Therefore, this study sought to compare perioperative and early postoperative opioid consumption in direct anterior approach THA patients following the use of a novel, fluoroscopic-assisted RA-THA system compared to a fluoroscopic-assisted, manual technique. The primary outcome of interest was the amounts of narcotics used by patients during their postoperative period.

## Methods

Institutional Review Board approval was obtained before the initiation of this study. We retrospectively reviewed the consecutive patients who underwent RA-THA in our institution from the study surgeon from September 2021 to July 2022. For comparison, we also reviewed all consecutive patients who received manual THA (mTHA) from the study surgeon from March 2021 to September 2021. Patients who met all of the following criteria were included in this study: (1) a preoperative diagnosis of osteoarthritis, avascular necrosis, or rheumatoid arthritis; (2) greater than 18 years of age; and (3) unilateral direct anterior THA from the primary study surgeon. Patients who met one of the following criteria were excluded (1) preoperative diagnosis of femoral neck fracture; (2) under 18 years of age; (3) bilateral THA; and (4) documented opioid use disorder and medical history of IV drug use.

### Opioid administration

Pain control regimens, including narcotic prescriptions, were standardized across both cohorts. All patients received preoperative single-shot spinal anesthesia, 60 cc local injections of marcaine intraoperatively, and 1 g of tranexamic acid on incision and 1 g on closure. Postoperatively, patients were prescribed acetaminophen 1,000 mg twice daily as needed for pain, aspirin 325 mg twice daily for deep vein thrombosis prophylaxis, and oxycodone 5 mg every 8 h as needed for breakthrough pain. In some elderly patients, oxycodone was occasionally substituted for tramadol 50 mg twice daily as needed. Additionally, patients received cyclobenzaprine 10 mg twice daily as needed for muscle spasms and icing three times daily. We queried the medication administration record and prescription drug monitor program (PDMP) database in electronic health records for baseline/preoperative opioid use, as well as the amounts of opioids given to each patient postoperatively. Additionally, we collected the amounts of narcotics prescribed at the time of discharge, any additional MME prescribed within the 6-week postoperative period, and any additional MME prescribed at the time of the 6-week postoperative follow-up appointment. These amounts were collected by querying the PDMP database in the electronic health record.

### Assessment

The average amount of narcotics in morphine milligram equivalents (MME) given to each cohort in-hospital was compared using data collected from the medication administration record, in addition to the amounts given in the post-anesthesia care unit (PACU) and on the hospital floor (if the patient was admitted). We also compared the amounts of postoperative opioids in MME prescribed after hospital discharge. The total average amount of postoperative narcotics in the 6-week postoperative period tabulated by summing the in-hospital and post-discharge amounts was compared between groups. Post-discharge narcotics included those prescribed at the time of discharge as well as those prescribed outpatient between the time of hospital discharge to the 6-week follow-up visit. The proportion of patients that received < 400 MME narcotics postoperatively was also compared [17]. Patient-reported pain was also measured using visual analog scale (VAS) pain scores (scaled from 0–10). Baseline scores were recorded from preoperative visits. The average in-hospital scores were collected from all pain scores recorded during patient hospital stays. Postoperative scores were collected during 6-week postoperative follow-up appointments. The 6-week postoperative period was selected to correspond with our postoperative management paradigm, as patients are regularly seen twice following their procedure, at 2 weeks and 6 weeks postoperatively.

We additionally performed a stratified analysis of these above outcomes based on preoperative opioid naivety. Preoperative opioid naivety was determined by querying the PDMP for patients’ Narx Scores, a numeric score between 0 and 999 that approximates the risk of accidental overdose based on a patient’s overall opioid usage [18, 19]. Patients with a preoperative Narx Score of zero were considered opioid naïve [20, 21]; any score higher than zero was considered opioid-tolerant for analysis stratification.

Patient characteristics and treatment data, including age at the time of the procedure, sex, race, body mass index (BMI), preoperative arthritis diagnosis, procedure laterality, American Society of Anesthesiologists (ASA) score, LOS, and percent of patients with an opioid prescription for chronic pain were also recorded and compared across treatment groups.

### RA-THA and mTHA

For the study cohort, a consecutive series of direct anterior approach THA was performed using a fluoroscopy-based RA-THA platform, the ROSA® Total Hip System (Zimmer CAS, Montreal, QC, Canada). The RA-THA workflow was adopted in September 2021 using a surgical workflow previously published by Kamath et al. [22]. Other than the use of robotic assistance, the surgical technique was identical for both study arms and was performed by the same surgical team. Prior to September 2021, the principal surgeon performed manual direct anterior approach THA with fluoroscopic guidance using a standard 12-inch C-arm for assistance with leveling the pelvis, bone preparation, and assessment of component position, which served as the control cohort. Perioperative recovery and pain management protocols were also identical for both study groups. No changes were made in perioperative physical therapy, recovery protocols, and surgical or clinical orthopedic team personnel during the study period.

### Statistical analysis

Baseline comparisons of patient demographics and treatment data between study groups were made. Continuous variables were reported as means and standard deviations (SD) and compared between groups using independent sample *t*-tests. Categorical variables were presented as frequencies and compared using Pearson’s chi-squared tests or Fisher’s exact tests when appropriate. Statistical analyses were performed using JMP Version 16.2*.* (SAS Institute Inc., Cary, NC, USA, 1989–2021). Based on previously reported narcotic consumption patterns following THA we sought to include approximately 100 patients per arm to detect a minimal clinically important difference of a relative 40% reduction of in-hospital postoperative narcotic assumption with 80% statistical power [23].

## Results

Against selection criteria, 211 patients were identified and included in our study sample: 104 patients underwent mTHA and 107 patients underwent RA-THA. There were no other significant differences in baseline patient characteristics or treatment data between cohorts (Table 1).

**Table 1:** Patient demographic, baseline treatment data, and disposition/discharge status between manual THA and robotic-assisted THA cohorts

	**Technique**		***P* value**
	**Manual THA**	**Robotic THA**	
	*n* = 104	*n* = 107	
Age at Surgery (Years)	60.0 (15.1)	60.5 (14.1)	0.813
Gender (% Female)	53%	47%	0.371
BMI	29.1 (5.1)	29.8 (4.9)	0.292
Race			
(% Caucasian)	80%	80%	0.596
(% Black)	20%	19%	
(% Other/ Multiracial)	0%	1%	
Side (% Left)	39%	47%	0.225
Preoperative Diagnosis			
(% Osteoarthritis)	87%	85%	0.611
(% Avascular Necrosis)	13%	13%	
(% Rheumatoid Arthritis)	0%	2%	
ASA Score			
(% Class I)	2%	1%	0.140
(% Class II)	48%	45%	
(% Class III)	46%	54%	
(% Class IV)	4%	0%	

Total number of patients *n* = 211

*BMI* Body Mass Index, *ASA Score* American Society of Anesthesiologists Score

**Fig. 1:**
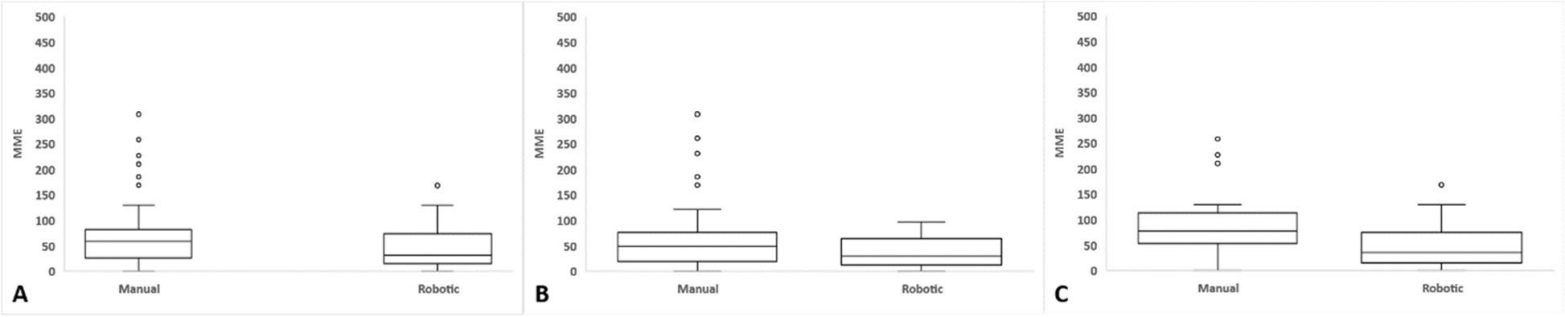
In-hospital narcotic usage patterns for (**A**) overall manual and robot-assisted cohorts, **B** opioid-naïve patients, and **C** opioid-tolerant patients

The RA-THA cohort had lower rates of opioid naivety compared to the manual group (62% vs. 79%; *P* = 0.008). Despite this, the RA-THA cohort had lower total in-hospital narcotics use compared to the mTHA cohort (42.3 vs. 66.4 MME; *P* < 0.001), and lower hospital floor narcotics use (29.1 vs. 55.0 MME; *P* < 0.001). These differences were similarly observed in both the opioid-naïve and opioid-tolerant groups in stratified analysis (Table 2). Of note, three patients in total had unclear opioid naivety (2 RA-THA and 1 mTHA). All of these patients were international and traveled to our institution for their procedures. As such, there was no prior information on their opioid naivety available in the PDMP. Therefore, these three patients were omitted from the opioid-naïve and opioid-tolerant sub-analyses. Box-and-whisker plots presenting in-hospital opioid use for patients who received RA-THA and mTHA are shown in Fig. 1.

**Table 2:** Baseline narcotic usage data and in-hospital narcotic usage patterns

	**Treatment**		***P* value**
	**Manual THA**	**Robotic THA**	
	*n* = 104	*n* = 107	
Opioid Naïve (% Yes)	79%	62%	**0.008**
Chronic Pain Prescriptions (% Yes)	1%	1%	0.984
In-hospital Narcotics			
Total (MME)	66.4 (59.5)	42.3 (33.9)	** < 0.001**
PACU (MME)	11.2 (12.8)	13.3 (10.9)	0.200
Hospital Floor (MME)	55.0 (51.9)	29.1 (31.4)	** < 0.001**
Opioid Naive	*n* = 81	*n* = 65	
In-hospital Narcotics			
Total (MME)	59.4 (56.4)	38.0 (30.1)	**0.004**
PACU (MME)	10.4 (12.8)	11.9 (10.4)	0.451
Hospital Floor (MME)	48.9 (47.9)	26.3 (29.2)	** < 0.001**
Opioid Tolerant	*n* = 22	*n* = 40	
In-hospital Narcotics			
Total (MME)	91.9 (66.4)	50.1 (39.1)	**0.011**
PACU (MME)	14.0 (13.4)	15.3 (11.7)	0.719
Hospital Floor (MME)	77.0 (61.8)	34.8 (34.6)	**0.006**

Significance bolded at a level of *P* < 0.05

*MME* Morphine Milligram Equivalents, *PACU* Post-Anesthesia Care Unit

**Fig. 2:**
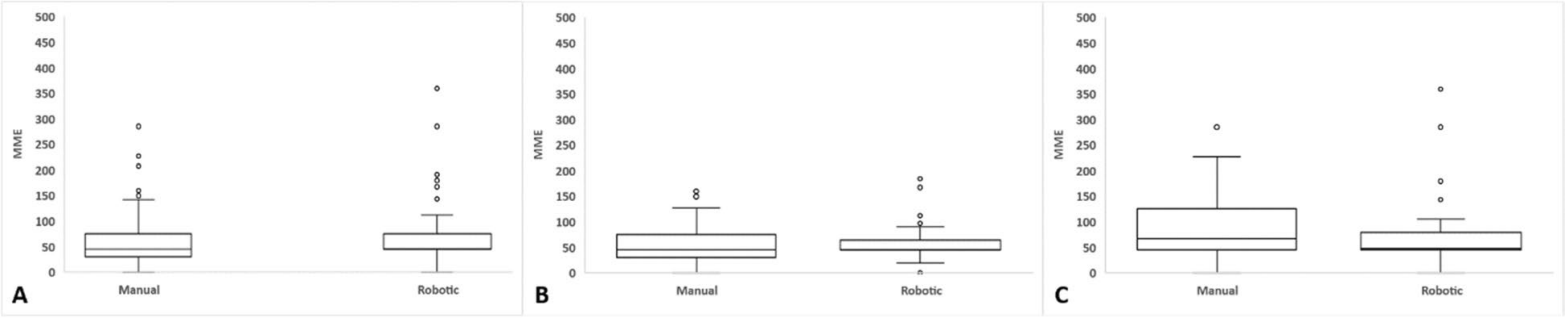
Post-discharge narcotic usage patterns for **A** overall manual and robot-assisted cohorts, **B** opioid-naïve patients, and **C** opioid-tolerant patients

No differences were detected in the amount of narcotics prescribed within the 6-week postoperative period, the proportion of patients that required prescription refills, or the average number of refills prescribed between the two groups (Table 3). Box-and-whisker plots presenting post-discharge opioid use for patients who received RA-THA and mTHA are shown in Fig. 2.

**Table 3:** Outpatient postoperative narcotic use up to 6 weeks after operation for manual and robotic-assisted cohorts

	**Treatment**		***P* value**
	**Manual THA**	**Robotic THA**	
	*n* = 104	*n* = 107	
Narcotics at Discharge (MME)	36.2 (15.2)	38.6 (16.2)	0.276
Additional Narcotics w/in 6-weeks			
Additional Narcotics (% Yes)	42%	39%	0.713
Number of Narcotics Refills	0.8 (1.4)	0.8 (1.4)	0.787
Additional Narcotics (MME)	22.1 (35.2)	21.9 (41.7)	0.980
Additional Narcotics at 6-week Follow-up			
Additional Narcotics (% Yes)	13%	11%	0.610
Additional Narcotics (MME)	4.1 (15.0)	2.4 (8.3)	0.296
Opioid Naïve	*n* = 81	*n* = 65	
Narcotics at Discharge (MME)	35.4 (15.0)	39.0 (13.4)	0.128
Additional Narcotics w/in 6-weeks			
Additional Narcotics (% Yes)	40%	31%	0.272
Number of Narcotics Refills	0.7 (1.0)	0.6 (1.3)	0.918
Additional Narcotics (MME)	17.4 (26.2)	15.5 (31.2)	0.689
Additional Narcotics at 6-week Follow-up			
Additional Narcotics (% Yes)	10%	9%	0.876
Additional Narcotics (MME)	1.8 (6.4)	1.3 (4.4)	0.582
Opioid Tolerant	*n* = 22	*n* = 40	
Narcotics at Discharge (MME)	39.5 (15.6)	38.0 (20.1)	0.751
Additional Narcotics w/in 6-weeks			
Additional Narcotics (% Yes)	50%	55%	0.706
Number of Narcotics Refills	1.5 (2.1)	1.1 (1.5)	0.385
Additional Narcotics (MME)	39.1 (55.0)	32.4 (53.4)	0.644
Additional Narcotics at 6-week Follow-up			
Additional Narcotics (% Yes)	23%	13%	0.295
Additional Narcotics (MME)	12.6 (28.8)	4.08 (12.1)	0.197

*MME* Morphine Milligram Equivalents

**Fig. 3:**
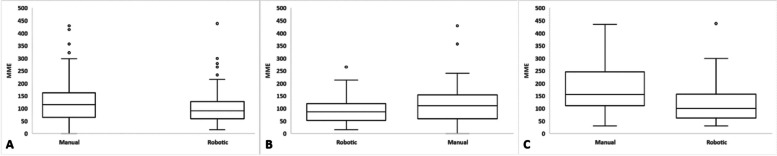
Total postoperative narcotic usage patterns up to 6 weeks postoperative for (**A**) overall manual and robot-assisted cohorts, **B** opioid-naïve patients, and **C** opioid-tolerant patients

When taken together, the RA-THA cohort had significantly lower total postoperative narcotics use compared to the mTHA cohort (103.7 vs. 127.8 MME; *P* = 0.025). This difference was similarly seen amongst opioid-tolerant patients (123.6 vs. 181.3 MME; *P* = 0.042), but we did not detect a significant difference amongst opioid-naïve patients (93.8 vs. 114.0 MME; *P* = 0.052) (Table 4). Box-and-whisker plots presenting total postoperative opioid use for patients who received RA-THA and mTHA are shown in Fig. 3. No differences were seen between groups with respect to the proportion of patients who received < 400 MME in the overall and stratified analyses (Table 4).

**Table 4:** Total postoperative narcotic use up to 6 postoperative weeks for manual and robotic-assisted cohorts

	**Treatment**		***P* value**
	**Manual THA**	**Robotic THA**	
	*n* = 104	*n* = 107	
Total Postoperative Narcotics (MME)	127.8 (87.1)	103.7 (66.6)	**0.025**
< 400 MME (%)	97%	99%	0.299
Opioid Naive	*n* = 81	*n* = 65	
Total Postoperative Narcotics (MME)	114.0 (74.0)	93.8 (49.8)	0.052
< 400 MME (%)	99%	100%	0.369
Opioid Tolerant	*n* = 22	*n* = 40	
Total Postoperative Narcotics (MME)	181.3 (111.8)	123.6 (84.3)	**0.042**
< 400 MME (%)	91%	98%	0.247

Significance bolded at a level of *P* < 0.05

*MME* Morphine Milligram Equivalents

Comparison of patient-reported pain scores showed no significant differences in baseline pain scores (6.4 vs. 6.1; *P* = 0.237), average in-hospital pain scores (4.8 vs. 4.9; *P* = 0.481), and 6-week postoperative pain scores (1.7 vs. 1.8; *P* = 0.762) between the RA-THA and mTHA study cohorts (Table 5).

**Table 5:** Patient-reported pain scores for manual and robotic-assisted cohorts

	**Treatment**		***P* value**
	**Manual THA**	**Robotic THA**	
	*n* = 104	*n* = 107	
Baseline	6.1	6.4	0.237
In-hospital	4.9	4.8	0.481
6-Week Post-op	1.8	1.7	0.762
Opioid Naïve	*n* = 81	*n* = 65	
Baseline	5.9	6.3	0.244
In-hospital	4.7	4.5	0.412
6-Week Post-op	1.9	2.0	0.737
Opioid Tolerant	*n* = 22	*n* = 40	
Baseline	6.9	6.8	0.907
In-hospital	5.6	5.2	0.471
6-Week Post-op	1.8	1.3	0.446

## Discussion

To our knowledge, this study presented the first findings of the postoperative narcotics burden associated with RA-THA. Our results demonstrated that patients who underwent RA-THA received fewer opioids during the 6-week postoperative period, consistent in both the overall analysis and opioid-tolerant analysis. While not statistically significant, likely due to lack of statistical power, the difference in total postoperative narcotics between groups for opioid-naïve patients was 20.2 MME. Our results also showed patients who received RA-THA received fewer narcotics during their immediate perioperative period and/or hospital stay compared to those who underwent mTHA, consistent across both opioid-naïve and opioid-tolerant patient subgroups. Although there was no statistical difference seen in the number of opioids prescribed after discharge, given that there was no corresponding increase in outpatient narcotic use, this decrease in the number of opioids consumed by RA-THA patients in-hospital cannot be discounted as changes in pain management strategy that occurred over the study period.

Importantly, our results also demonstrated statistically equivalent patient-reported pain between the two groups throughout the treatment course. Despite advancements in surgical technique and contemporary multimodal pain regimens that have reduced postoperative pain, significant pain can still be anticipated following the procedure, with the average in-hospital VAS pain postoperative pain being reported to be 4/10 even with the administration of basic perioperative analgesics [1, 14, 24]. This suggests that patients treated with mTHA required greater amounts of postoperative narcotics to achieve comparable levels of pain control as those treated with RA-THA. The mechanism by which the RA-THA system reduced postoperative opioid requirements remains to be elucidated. While it is possible that the RA-THA cohort requested fewer as-needed narcotics for breakthrough pain than the mTHA cohort, the similarity of the baseline characteristics of these groups, lower opioid naïvety amongst RA-THA patient, and similar postoperative pain profiles makes this explanation less likely. This RA-THA system has been previously shown to improve the accuracy of acetabular cup positioning compared to manual techniques [25]. Similar reductions in postoperative opioid consumption following robotic-assisted total knee arthroplasty (RA-TKA) have been attributed to improved accuracy associated with robotic-assisted arthroplasty systems that improve restoration of native biomechanics and reduce soft tissue release [12, 26]. It is possible that improved component placement with the RA-THA system similarly results in less intraoperative soft tissue trauma and improves restoration of native hip biomechanics that translates to lower postoperative pain, faster recovery, and subsequent lower narcotic burden.

While our results are difficult to contextualize due to the absence of comparative studies in the current literature investigating the relationship between postoperative opioid burden and RA-THA, it is important to place our findings in the context of what has been published for other direct anterior approach THA protocols. Guidelines put forth by the AAOS and adopted by the Mayo Clinic in 2017 called for a maximum MME of 400 for total hip arthroplasty within 12 weeks of the procedure [17]. While our present study only investigated postoperative opioid use up to 6-weeks following procedures, the average total MME following RA-THA in this study was significantly below this threshold, with > 99% of RA-THA receiving < 400 MME, including 98% of opioid-tolerant patients. Likewise, several teams have investigated the relationship between surgical approach and postoperative opioid use. Seah et al. found that patients who received mTHA with the direct anterior approach had significantly less in-hospital opioid usage than patients who received mTHA with the lateral, anterolateral, or posterolateral approaches (63.05 ± 42.97 vs. 79.81 ± 56.10 vs. 77.50 ± 54.52 MME/day; *P* < 0.05) [27]. In contrast, Bovonratwet et al. found that direct anterior approach THA was not associated with a statistically significant reduction in total inpatient MME consumed compared to a posterior approach (79.8 vs. 100.1, *P* = 0.486), or in opioid prescription refill within 3 months after discharge (15% vs. 21% *P* = 0.864) [28]. Although direct comparisons are difficult to make, it is important to note that the total in-hospital opioid use for direct anterior approach THA patients in this study was less than that reported for our RA-THA group, but comparable to that of our mTHA cohort, further validating our results.

Additionally, although the postoperative opioid burden associated with RA-THA remains to be elicited in the current literature, several research groups have reported lower postoperative narcotic burden for patients undergoing RA-TKA compared to manual techniques [12, 13, 15, 29, 30]. Bhimani et al. reported that patients undergoing RA-TKA required 3.2 mg fewer morphine equivalents per day during the 6-week postoperative period compared to patients treated with manual TKA (*P* < 0.001) [12]. Similarly, Ofa et al. reported higher total MME in the 90-day postoperative period for their manual TKA cohort compared to the RA-TKA cohort (1150 MME vs. 873 MME; *P* < 0.001) [15]. In contrast, in their matched analysis, Samuel et al. detected no difference in median in-hospital opioid consumption between patients who underwent RA-TKA and those who underwent manual TKA (60.0 vs. 70.0 MME/day; *P* = 0.57) [31]. Again, while it is difficult to draw direct comparisons to our results, the introduction of these robotic assistance platforms appears to reduce postoperative opioid use following total joint arthroplasty, but these findings require further exploration.

The strength of this study is that this is the first investigation of postoperative narcotic consumption patterns following RA-THA. Additionally, this study examined both in-hospital and post-discharge opioid use, which allows for more precise comparisons of the timing of postoperative use between treatment cohorts. Lastly, the inclusion of stratified analyses based on opioid naïvety provides further granularity of the differences in narcotic use between treatment arms based on patients’ previous opioid exposure.

Our study has several limitations. First, the study is subject to data extraction from the EHR. We mitigated this bias by collecting opioid amounts that were digitally recorded in the medication administration record and the PDMP. Second, as this was a retrospective study, outpatient opioid use was determined using the amount that was prescribed, rather than the amount consumed, thus could over-predict the true amounts of opioid patients consumed in the post-discharge period. Third, our unblinded pain management weakened the testing standards even though the same protocol was chosen by the same surgical team. Fourth, as the RA-THA cohort included the first fluoroscopy-based RA-THA cases performed by the study surgeon, it is possible that there was a learning curve effect for postoperative pain control. However, it has been previously reported that no learning curve effect was seen with this robotic system with respect to cup placement [32], thus it is unlikely that a learning curve exists with respect to postoperative pain control.

## Conclusion

The results of our present study demonstrated that fluoroscopy-based RA-THA is associated with lower 6-week postoperative opioid use, including during the immediate perioperative period, when compared to manual techniques, consistent for both opioid-naive and opioid-tolerant patients. Taken together, our findings can be used by surgeons to support the adoption of fluoroscopy-based RA-THA into their clinical practice given the low amounts of opioid consumption in the early postoperative period and no increased usage of narcotics within 6-weeks of surgery.

## Data Availability

The datasets used and/or analyzed during the current study are available from the corresponding author on reasonable request.
